# ISGylation Inhibits an LPS-Induced Inflammatory Response via the TLR4/NF-κB Signaling Pathway in Goat Endometrial Epithelial Cells

**DOI:** 10.3390/ani11092593

**Published:** 2021-09-03

**Authors:** Jinbang Xiao, Shanshan Li, Ruixue Zhang, Zongjie Wang, Xinyan Zhang, Aihua Wang, Yaping Jin, Pengfei Lin

**Affiliations:** 1 College of Veterinary Medicine, Northwest A&F University, Yangling 712100, China; 15680830507@nwafu.edu.cn (J.X.); lishanshan1003@nwafu.edu.cn (S.L.); zhangruixue@nwafu.edu.cn (R.Z.); wangzongjie@nwafu.edu.cn (Z.W.); xinyanzhang@nwafu.edu.cn (X.Z.); aihuawang1966@163.com (A.W.); 2 Key Laboratory of Animal Biotechnology, Ministry of Agriculture and Rural Affairs, Northwest A&F University, Yangling 712100, China

**Keywords:** ISGylation, endometritis, inflammatory, TLR4/NF-κB, goat

## Abstract

**Simple Summary:**

Endometritis is a common and important reproductive disease of domestic animals, leading to repeated infertility, abortion, and ovarian dysfunction, which affects the reproductive rate and production performance of female domestic animals, and causes serious financial loss to farmers. Infection with Gram-negative bacteria, the release of LPS and activation of the TLR4/NF-κB signaling pathway are the principal factors responsible for the disease. However, the mechanism of the interaction between endometrial immunity and bacterial infection is not entirely clear. Ubiquitin-like protein ISG15 can regulate the TLR4/NF-κB signaling pathway via the ISGylation modification system, which modulates the inflammatory response. In the present study, we found that ISG15 proteins were mainly located in the cytoplasm of goat endometrial epithelial cells (gEECs) and that the expression of key genes and proteins of ISGylation increased in LPS-induce gEECs. Overexpression and silencing of the ISG15 gene demonstrated that ISGylation inhibited an LPS-induced inflammatory response via the TLR4/NF-κB signaling pathway in gEECs. Here, we provide the experimental basis for further exploration of the role of the ISGylation modification system in the inflammatory response of endometrium and a potential method for the treatment of endometritis.

**Abstract:**

Endometritis is a common and important reproductive disease of domestic animals. The principal factors responsible for the disease are infection with Gram-negative bacteria, the release of Lipopolysaccharides (LPS) and activation of the TLR4/NF-κB signaling pathway. However, we do not fully understand the interaction between endometrial immunity and bacterial infection in the disease etiology. The ubiquitin-like protein ISG15 can regulate the TLR4/NF-κB signaling pathway via the ISGylation modification system, modulating the inflammatory response. In the present study, we found that ISG15 protein was expressed mainly in the cytoplasm of goat endometrial epithelial cells (gEECs) and that the expression of key genes and proteins of ISGylation increased in LPS-induced gEECs. Overexpression and silencing of the *ISG15* gene demonstrated that ISGylation inhibited an LPS-induced inflammatory response via the TLR4/NF-κB signaling pathway in gEECs. Here, we provide the experimental basis for further exploration of the role of the ISGylation modification system in the inflammatory response of endometrium and a potential method for the treatment of endometritis.

## 1. Introduction

Endometritis is a common reproductive disease in domestic animals. It may lead to repeated infertility, abortion, and ovarian dysfunction, affecting the reproductive rate and thus production in domestic animals, which can cause serious financial loss to farmers [1,2]. Pathogenic microorganisms infecting the uterus, either singly or in combination, release virulence factors and cause uterine tissue damage and inflammatory reactions. This is the principal cause of endometritis [3], which results mostly from infections from bacteria, such as *Escherichia coli*, *Trueperella pyogenes*, *Clostridium*, *Streptococcus*, or *Staphylococcus* [4,5,6]. Lipopolysaccharides (LPS), the main component of the cell wall of Gram-negative bacteria, is an important virulence factor that causes endometritis, provoking a robust inflammatory response in the uterus of female domestic animals. Although there have been many reports of LPS-induced uterine inflammation [7,8,9], the endometritis mechanism by which LPS-induced in ruminants is still not entirely clear.

The development of endometritis depends on the virulence of the pathogenic microorganisms and their ability to overcome uterine tolerance and resistance to infection. The endometrium is the first line of defense against pathogen invasion and initiates both an innate and adaptive immune response following infection. Type I interferon induces hundreds of interferon-stimulating genes (ISGs) to regulate the stress response [10], which play core roles in regulating the host’s immune response to pathogens. Of the ISGs, ISG15 produces the strongest and most rapid response [11]. ISG15 is a ubiquitin-like protein and binds covalently to cellular or pathogen-associated substrates, modifying them by ISGylation under the action of E1(activating enzyme, UBE1L)-E2(conjugating enzyme, UBE2L6)-E3(binding enzyme, HERC5/6 or TRIM25) reactions [12]. In addition to interferon, LPS, foreign DNA or RNA, retinoic acid, and DNA damaging agents also can induce the expression of ISG15 and the activation of the ISGylation modification system [13], thus playing a regulatory role in various biological processes such as immune regulation [14], anti-viral protection [15], and prevention of nerve injury [16] and cancer [17]. Studies have shown that LPS promotes the release of free ISG15 from monocytes and lymphocytes [18], inducing high expression levels of ISG15 and activation of ISGylation in macrophages [19], suggesting that ISGylation is closely associated with inflammation. The microarray results have shown that ISG15 in mixed bovine epithelial and stromal endometrial cells was significantly upregulated after being exposed to LPS [20]. However, the role of ISGylation in endometritis remains to be explored.

The TLR4/NF-κB signaling pathway operates in many types of eukaryotic cells and can be activated by LPS, bacterial glycoproteins, viral molecules, and inflammatory factors, leading to the regulation of cellular activity in the LPS-induced inflammatory response [21]. The TLR4/NF-κB signaling pathway is the principal pathway activated in the LPS-induced inflammatory response and forms an important element of the endometrial innate immune system [3]. Inhibition of TLR4/NF-κB signaling to reduce LPS-induced endometritis has recently been explored in the study of disease pathogenesis and the development of therapeutic drugs [22,23]. It also has been reported that ISG15 inhibits the NF-κB signaling pathway by downregulation of IKKβ and p65 expression, in addition to phosphorylation of p65 and IκBα [24], suggesting that the ISGylation system may play a regulatory role through this signaling pathway. However, to date, its role in goat endometritis has not been explored. Therefore, in the present study, goat endometrial epithelial cells (gEECs) were initially activated by the LPS to establish a goat endometritis cell model to explore the localization and expression of key components of the ISGylation modification system. We then silenced and overexpressed ISG15 in the cells to explore the role of the ISGylation modification system in LPS-induced endometritis by regulation of the TLR4/NF-κB signaling pathway. The purpose of the present study was to lay a foundation and provide data to support the investigation of ISGylation and similar ubiquitin-like modification systems in the pathogenesis of endometritis.

## 2. Materials and Methods

### 2.1. Ethics Statement and Consent to Participate

The present study experiments were conducted under the strict guidelines of the Committee for the Ethics on Animal Care and Experiments in Northwest A&F University.

### 2.2. Cell Culture and LPS Treatment

The gEECs were established by transfection with human telomerase reverse transcriptase (hTERT) and were well preserved in our laboratory [25]. gEECs were seeded in six-well plates and cultured with DMEM/F12 medium (HyClone, South Logan, UT, USA) containing 10% fetal bovine serum (FBS) (Corning, Manassas, VA, USA) at 37 °C in 5% CO_2_. After reaching 70%–80% confluence, the cells were treated with 5 μg/mL LPS from *Escherichia coli* 0111: B4 (3050; Sigma-Aldrich, Co., St. Louis, MO, USA) for 0, 3, 6 and 12 h. Three independent experiments were performed unless stated otherwise. At the end of the culture, the cells were collected for mRNA and protein experiments. The concentration of LPS was determined by previous studies in our laboratory and has been proved to cause an obvious inflammatory response of gEECs [26].

### 2.3. Overexpression Plasmid Transfection

Overexpression plasmids of ISG15 were constructed using pcDNA3.1(+) vectors, such as pcDNA3.1-ISG15. Empty plasmids were used as negative controls. The sequences of the primers used for the amplification of ISG15 complementary DNA (cDNA) fragments were as follows: forward primer 5′-cgggatcctgacaccagaacccacggcc-3′ and reverse primer 5′-ccctcgagtaaggccctcctggcccttccc-3′.

The gEECs were cultured at densities of 1 × 10^5^ cells per mL in Dulbecco’s Modified Eagle Medium (DMEM)/F12 supplemented with 10% FBS in 6-well culture plates. After reaching 70–80% confluence, the gEECs were transfected with the appropriate overexpression plasmids using the TurboFect transfection reagent (Thermo Fisher Scientific, Waltham, MA, USA), in accordance with the manufacturer’s protocol. After transfection for 12 h, the culture media was exchanged for fresh medium, and incubation was continued for a further 12 h. The transfected cells were then treated with 5 μg/mL LPS for 12 h prior to analysis in the following experiments. Three independent experiments were performed unless stated otherwise. 

### 2.4. ISG15 siRNA Interference Fragment Design and Transfection

ISG15 siRNA interference fragments were constructed by Shanghai Ji Ma Pharmacy Technology Co., Ltd. (Ji Ma, Shanghai, China). A si-NC sequence fragment, without any specific target, was also synthesized as a negative control. The ISG15 siRNA sequence is showed in Table 1. The detailed method of transfection is described in Section 2.3, except that the gEECs were transfected with the appropriate ISG15 siRNA fragment plasmids using the TurboFect transfection reagent.

### 2.5. qRT-PCR Analysis

The total RNA was extracted from uterus samples using TRIzol reagent (Invitrogen, Inc., Carlsbad, CA, USA). After reverse transcription into cDNA by the 5X All-In-One RT MasterMix with AccuRT Genomic DNA Remove Kit (Applied Biological Materials Inc., Vancouver, BC, Canada), qRT-PCR was performed using an EvaGreen qPCR Mastermix Kit (AceQ^®^Universal SYBR^®^qPCR Master Mix. Vazyme Biotech Co., Ltd, Nanjing Jiangsu, China) according to the manufacturer’s instructions in the CFX96™ Real-Time PCR Detection System (Bio-Rad Laboratories, Inc., Hercules, CA, USA). The mRNA expression levels were normalized to the expression of GAPDH and quantified using the 2^−^^ΔΔCt^ method. All reactions were conducted in biological triplicate. The information of primers is listed in Table 2.

### 2.6. Western Blot Analysis 

The gEECs were lysed using a Total Protein Extraction Kit, and then the total protein was measured by the bicinchoninic acid (BCA) assay (Nanjing Keygen Biotech Co., Ltd., Nanjing, China). For each sample, 20 μg of total protein were separated using 15% sodium dodecyl sulfate-polyacrylamide gel electrophoresis (SDS-PAGE) followed by electro transfer to PVDF membrane (Millipore, Billerica, MA, USA). After blocking with 8% non-fat milk in TBST for 2 h, the membranes were incubated overnight at 4 °C with primary antibodies against ISG15 (CST, #15981-1AP; 1:1000 dilution), UBE1L (Proteinch, #YT4797; 1:1000 dilution), NF-κB p65 (GST, #8242; 1:1000 dilution), NF-κB p65(S536) (GST, #3033; 1:1000 dilution) or β-actin (1:2000; Beijing CWBIO Co., Ltd., Beijing, China). After the membranes were incubated with the secondary antibodies conjugated to HRP (1:5000 dilution; Zhong Shan Golden Bridge Biotechnology, Nanjing, China) for 1 h at room temperature, protein bands were detected using the SuperSignalWest Pico kit (Thermo, Rockford, IL, USA) in the Gel imaging system (Tanon-4100, Tanon Science & Technology Co., Ltd., Shanghai, China). The densitometric analyses were processed using ImageJ software (Bio-Rad).

### 2.7. Immunofluorescence

After treatment, the cells were fixed in 4% (wt/vol) paraformaldehyde for 30 min at room temperature, after which they were permeabilized using 0.1% Triton X-100 in phosphate-buffered salina (PBS) for 10 min. Cells were then blocked in 0.1% bovine serum albumin (BSA) in PBS for 1 h and then incubated overnight with primary antibodies at 4 °C, followed by three PBS washes for 5 min each time. The cells were incubated for 2 h with a red fluorescent-conjugated donkey anti-rabbit secondary antibody (1:200 dilution) at room temperature, followed by three PBS washes for 5 min each time. Coverslips were mounted over the cells on glass slides using ProLong Gold Antifade reagent (Thermo Fisher Scientific), with or without DAPI for visualization of cell nuclei. Images were acquired using a confocal microscope and post-processed in Adobe Photoshop for specific inset enlargement and RGB channel separation.

### 2.8. Statistical Analysis

All experiments were replicated at least three times for each group. Data were expressed as means ± SEM. The data were analyzed with one-way ANOVA followed by Tukey’s post hoc test and the Independent-Samples *T*-test using the GraphPad Prism 6 Software. Differences were considered significant when *p* was < 0.05.

## 3. Results

### 3.1. Expression of ISGylation on LPS-Induced Inflammatory Response in gEECs

**Figure 1:**
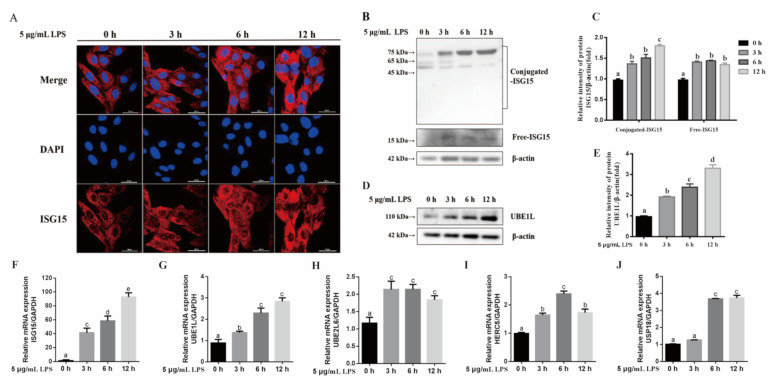
The expression of ISGylation on LPS-induced inflammatory response in gEECs. (**A**) The immunofluorescence of ISG15 after gEECs treated with 5 μg/mL LPS for 0, 3, 6 or 12 h. (**B**) Western blot analysis for conjugated and free ISG15 after gEECs treated with 5 μg/mL LPS for 0, 3, 6 or 12 h. (**C**) The quantification of ISG15 band intensities from three independent results was determined by densitometric analysis. (**D**) Western blot analysis for UBEL1 protein after gEECs treated with 5 μg/mL LPS for 0, 3, 6 or 12 h. (**E**) The quantification of UBEL1 band intensities from three independent results was determined by densitometric analysis. (**F**–**J**) The mRNA levels of ISG15, UBE1L, UBE2L6, HERC5, and USP18 after gEECs treated with 5 μg/mL LPS for 0, 3, 6 or 12 h, respectively. The data are presented as the mean ± SEM from three independent experiments, and bars with different letters are significantly different (*p* < 0.05).

To analyze the potential function of ISGylation in LPS-mediated gEECs inflammatory response, the expression levels of ISG15 and the key enzymes were examined (Figure 1). ISG15 protein was mainly localized in the cytoplasm of gEECs, and the expression levels of ISG15 protein were significantly upregulated with increasing time of 5 μg/mL LPS treatment (Figure 1A). The free ISG15 protein levels were significantly increased after treatment with 5 μg/mL LPS for 3 h, although no significant difference was observed from 3 h to 12 h (Figure 1B,C). In contrast, the levels of conjugated ISG15 protein significantly increased with time after treatment with 5 μg/mL LPS (Figure 1B,C, *p* < 0.05; Supplementary Materials Figure S1). Meanwhile, the levels of UBE1L protein were significantly upregulated with increasing time of LPS treatment (Figure 1D,E, *p* < 0.05). The qRT-PCR results further revealed that the ISG15 and UBE1L mRNA levels were significantly upregulated with increasing time of LPS treatment (Figure 1F,G, *p* < 0.05). The expression levels of UBE2L6 mRNA were significantly upregulated after treatment with 5 μg/mL LPS for 3 h (Figure 1H, *p* < 0.05). The mRNA levels of HERC5 were significantly elevated after treatment with 5 μg/mL LPS, and the peaking levels were observed at 6 h (Figure 1I, *p* < 0.05). Significant differences in USP18 mRNA levels were observed after treatment with 5 μg/mL LPS for 6 h (Figure 1J, *p* < 0.05).

### 3.2. Effect of ISG15 on LPS-Induced Inflammatory Response in gEECs

**Figure 2:**
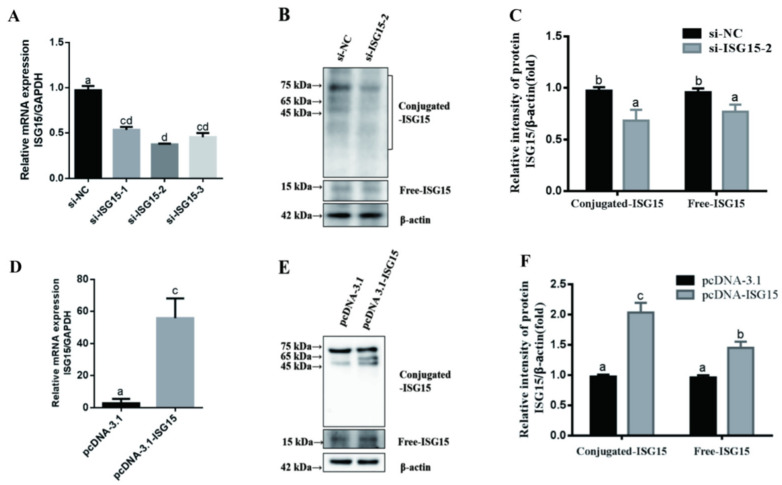
Efficiency verification of siRNA and overexpression ISG15 in gEECs. The gEECs were transduced with si-NC, si-ISG15, pcDNA-3.1, and pcDNA-ISG15 for 24 h, and then treated with 5 μg/mL LPS for 12 h, respectively. The efficiency of ISG15 silencing and overexpression were determined using qRT-PCR (**A**,**D**) and Western blot (**B**,**C**,**E**,**F**), respectively. The data are presented as the mean ± SEM from three independent experiments, and bars with different letters are significantly different (*p* < 0.05).

**Figure 3:**
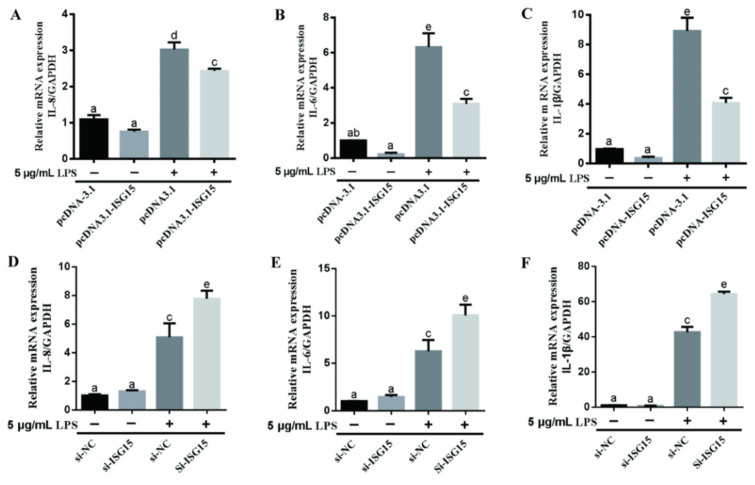
The effect of ISG15 on LPS-induced changes of inflammatory factors in gEECs. (**A**–**C**) qRT-PCR analysis of the mRNA levels of IL-8, IL-6, and IL-1β in 5 μg/mL LPS-treated gEECs for 12 h after transducing the overexpression vector pcDNA-ISG15, respectively. (**D**–**F**) qRT-PCR analysis of the mRNA levels of IL-8, IL-6, and IL-1β in 5 μg/mL LPS-treated gEECs for 12 h after transducing the si-ISG15, respectively. The data are presented as the mean ± SEM from three independent experiments, and bars with different letters are significantly different (*p* < 0.05).

To further reveal the role of ISGylation in LPS-induced inflammatory response, the gEECs were transfected with the siRNA or the overexpression vector pcDNA-ISG15 (Figure 2), and then the expression levels of IL-8, IL-6, and IL-1β were evaluated by qRT-PCR (Figure 3). As expected, both free and conjugated ISG15 protein expressions were significantly suppressed after transducing the si-ISG15, respectively (Figure 2A–C, *p* < 0.05; Supplementary Materials Figure S2). Meanwhile, transfection of the overexpression vector pcDNA-ISG15 in gEECs significantly upregulated the free and conjugated ISG15 protein expression, respectively (Figure 2D–F, *p* < 0.05; Supplementary Materials Figure S3). We found that overexpression ISG15 significantly inhibited IL-8, IL-6, and IL-1β expression in LPS-treated gEECs (Figure 3A–C, *p* < 0.05). Conversely, silencing of the ISG15 gene significantly upregulated IL-8, IL-6, and IL-1β expression in LPS-treated gEECs (Figure 3D–F, *p* < 0.05).

### 3.3. Effects of ISG15 on LPS-Induced TLR4/NF-κB Pathway in gEECs

**Figure 4:**
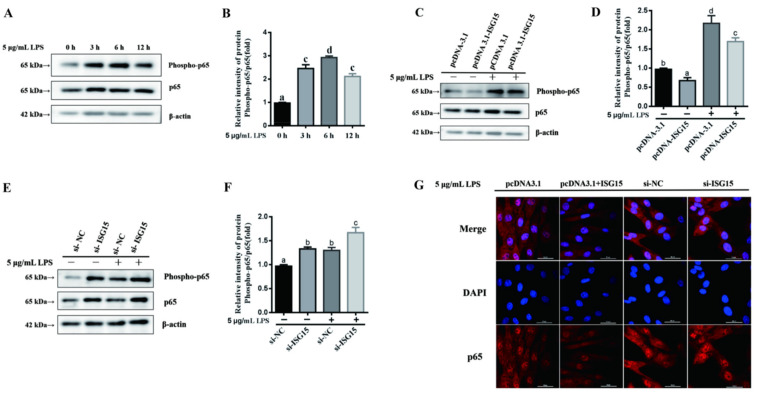
The effects of ISG15 on LPS-induced phospho-p65 in gEECs. (**A**,**B**) Western blot analysis of the phosphorylation levels of p65 after 5 μg/mL LPS-treated gEECs for 0, 3, 6 and 12 h, respectively. (**C**,**D**) Western blot analysis of the phosphorylation levels of p65 in 5 μg/mL LPS-treated gEECs for 12 h after transducing the overexpression vector pcDNA-ISG15. (**E**,**F**) Western blot analysis of the phosphorylation levels of p65 in 5 μg/mL LPS-treated gEECs for 12 h after transducing the si-ISG15. (**G**) The immunofluorescence of p65 in 5 μg/mL LPS-treated gEECs for 12 h after transducing the overexpression vector pcDNA-ISG15 or the si-ISG15, respectively. The data are presented as the mean ± SEM from three independent experiments, and bars with different letters are significantly different (*p* < 0.05).

**Figure 5:**
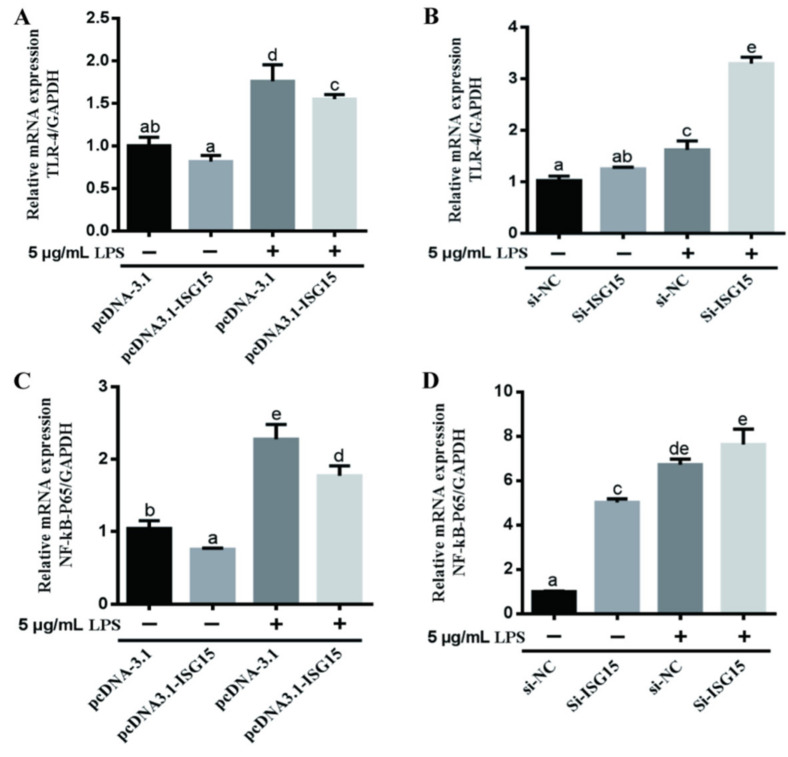
The effects of ISG15 on LPS-induced TLR4 and NF-κB-p65 in gEECs. qRT-PCR analysis of the mRNA levels of TLR4 (**A**,**C**) and NF-κB-p65 (**B**,**D**) with or without 5 μg/mL LPS-treated gEECs for 12 h after overexpression or silencing of ISG15, respectively. The data are presented as the mean ± SEM from three independent experiments, and bars with different letters are significantly different (*p* < 0.05).

To detect the anti-inflammatory mechanism of ISGylation, the expression of phospho-p65, TLR4 and NF-κB were measured. As shown in Figure 4A,B, the phosphorylation levels of p65 were significantly elevated after 5 μg/mL LPS treatment, with a peak at 6 h (*p* < 0.05). However, overexpression of ISG15 significantly suppressed the phosphorylation levels of p65 induced by LPS (Figure 4C,D, *p* < 0.05). Conversely, the phosphorylation levels of p65 induced by LPS were significantly upregulated after silencing of ISG15 in gEECs (Figure 4E,F, *p* < 0.05). Meanwhile, the immunofluorescence result demonstrated that the level of p65 in the nucleus induced by LPS was significantly downregulated and upregulated after knockdown or overexpression of the ISG15 gene, respectively (Figure 4G). The expression levels of TLR4 and NF-κB-p65 mRNAs induced by LPS were significantly repressed after ISG15 overexpression (Figure 5A,C, *p* < 0.05), and knockdown of ISG15 significantly promoted the mRNA levels of TLR4 and NF-κB-p65 (Figure 5B,D, *p* < 0.05).

## 4. Discussion

ISG15 represents the first ubiquitin-like modifier gene to be identified [27] and is widely found in mammalian immune and non-immune cells [27,28], constituting a modification system termed ISGylation, that uses a three-step enzymatic reaction similar to that of ubiquitination [29], while the deubiquitination enzyme USP18 removes ISG15 from target protein, thus reversing ISGylation [30]. ISG15 regulates cytokine secretion and immune cell activation through ubiquitination or in its free form [31]. In domestic animals, ISG15 has been shown to be expressed in epithelial and stromal cells of the endometrium in cattle, horses, and sheep [32,33,34] and is located in the nucleus, nuclear stroma, cytoplasm, and organelles of bovine subcellular structures [32]. In the present study, LPS was used to induce an inflammatory response in gEECs to successfully construct an in vitro model of goat endometritis, as has been previously reported in earlier studies [26]. Here, we found that ISG15 was mostly localized in the cytoplasm, with low levels of expression in the nucleus, essentially consistent with previous studies. The ISG15 expression was significantly increased in a non-type І interferon-dependent manner after LPS induction. As the time after LPS treatment increased, there was an upward trend in the level of conjugated ISG15, while the levels of free ISG15 did not change significantly, suggesting that ISG15 may preferentially react via ISGylation. In certain cells, ISG15 and ISGylation related enzymes are mainly expressed in the nucleus [35]; therefore, to further explore ISGylation activation, we investigated the mRNA levels of ISG15, UBE1L, UBE2L6, HERC5 and USP18, observing that they were significantly upregulated after 6 h of LPS treatment, with changes in the UBE1L protein levels consistent with the mRNA expression. These results are consistent with those of Piotr Przanowski [36], suggesting that ISGylation plays a regulatory role in the process of LPS-induced goat endometritis.

Although ISG15 and ISGylation play important roles in both immunity and inflammation [37], the majority of studies have focused on the induction of type І interferon, with few reports exploring the LPS-induced inflammatory response. ISG15 was found to be upregulated in the endometrium after perfusion of LPS into bovine mammary glands [38], while endometrial and stromal cells infected with bovine viral diarrhea virus (BVDV) reduced the response of ISG15 to LPS [39], suggesting the involvement of ISG15 in the regulation of inflammation and the immune response in endometritis; ISGylation increases the stability of numerous proteins including signal transducer and activator of transcription 1 (STAT1), preventing the premature termination of the immune response in LPS-stimulated microglia, and playing a protective role in inflammation [36]. In the present study, the overexpression of ISG15 significantly reduced the mRNA expression levels of IL-1β, IL-6 and IL-8 in gEECs following LPS treatment, while the silencing of ISG15 had the opposite effect, indicating that ISG15 plays a protective role in goat endometritis. These results are contrary to Jun-Bao’s findings that type I interferon induces the expression of cytokines involved in ISGylation and intensifies colon inflammation in mice [40]; the discrepancy may be related to factors, such as cell type, inflammation type, induction method, and time.

Innate immunity is closely associated with the mechanisms of recovery after delivery in ruminants [41], and the TLR4/NF-κB signaling pathway is known to activate in *E. coli*-induced endometritis. Studies have reported the use of hormones [23,42], natural product extracts [22,43], functional proteins [44,45], trace elements [46] and microRNA [47,48] that regulate the TLR4/NF-κB signaling pathway to alleviate endometrial inflammation. ISG15 induces cancer cell death through inhibition of NF-κB signaling [24] and is positively correlated with IκBα or phosphorylated IκBα in ovarian high-grade serous carcinoma. In the present study, the addition of LPS to gEECs upregulated the mRNA expression levels of TLP4 and NF-κB and significantly increased the phosphorylation of p65, indicating activation of the TLR4/NF-κB signaling pathway. Overexpression and interference with ISG15 significantly downregulated and upregulated the mRNA expression levels of TLP4 and NF-κB, respectively. Furthermore, overexpression of ISG15 decreased p65 phosphorylation and nucleation, while interference with ISG15 expression had the opposite effect, suggesting that ISG15 may play a role in alleviating inflammation by inhibition of the TLR4/NF-κB signaling pathway in LPS-induced endometritis. A number of studies have reported that IFN-τ alleviates endometritis by inhibiting NF-κB [44,49,50], possibly by inducing the overexpression of multiple ISGs, including ISG15.

In ruminants, IFN-τ is an important maternal gestation-recognition signaling factor secreted by embryonic trophoblast cells [51], that is highly expressed during embryo implantation and stimulates significant upregulation of ISG15 [52]. The establishment of pregnancy requires transient regulation of innate and adaptive maternal immunity [53]. The results of the present study demonstrated that ISG15 inhibited the TLR4/NF-κB signaling pathway, and such temporary immune tolerance may lead to weakened identification of pathogenic microorganisms in the body during this period, thus increasing the risk of infection. However, type I interferon mainly mediates the upregulation of ISG through the JAK/STAT signaling pathway [54], and so the interaction between the multiple signaling pathways still requires additional exploration.

## 5. Conclusions

We found that ISG15 protein expression was mainly located in the cytoplasm of gEECs, and ISGylation was increased in a time-dependent manner after LPS induction. ISGylation inhibited LPS-induced inflammatory responses via the TLR4/NF-κB signaling pathway in gEECs. The results provide an experimental basis for further exploring the role of the ISGylation modification system in the inflammatory response of the endometrium.

## Figures and Tables

**Table 1:** The information of the siRNA sequence.

si RNA Name	Sequences (5′-3′)
ISG15 siRNA-1	F: GGACCAAUUCUGGCUGUCUTT
	R: AGACAGCCAGAAUUGGUCCTT
ISG15 siRNA-2	F: GGACUCCAUGAUGGUAUCUTT
	R: AGAUACCAUCAUGGAGUCCTT
ISG15 siRNA-3	F: UCCUGCUGAUGGUGCAGAATT
	R: UUCUGCACCAUCAGCAGGATT
si-NC	F: UUCUCCGAACGUGUCACGUTT
	R: ACGUGACACGUUCGGAGAATT

**Table 2:** Primer sequences for qRT-PCR.

Target Gene	Primer Sequence (5′-3′)	Product Size(bp)
IL1β	F: TCCACCTCCTCTCACAGGAAA	99
(XM_013967700.2)	R: TACCCAAGGCCACAGGAATCT	
IL-6	F: CCTCTTCACAAGCGCCTTCA	122
(NM_001285640.1)	R: TGCTTGGGGTGGTGTCATTC	
IL-8	F: CTGGCCAGGATTCACGAGTT	177
(XM_005681749.3)	R: TGCTTCCACATGTCCTCACA	
ISG15	F: GAGATCCTGGTGCCTCTGAG	220
(XM_005690795.3)	R: CTACCAGGATGTTCAGGGT	
UBE1L	F: CCCTGGCATTCTCACTCTGA	213
(XM_005695948.3)	R: TGGCTCTTGACCTCAGTG	
UBE2L6	F: GGTGGCGAAGGAGAGGC	124
(XM_ 018059059.1)	R: CACGTTGGGGTGGTAGATCC	
HERC5	F: GATAGCATGTGGAAGGCAGC	210
(XM_018049314.1)	R: TGATTCCCTCCAGCAACCAT	
USP18	F: CCATTGTTTGTCCAGCACGA	231
(XM_018048313.1)	R: CAGTGTTTTCAGGGGCTTCC	
TLR4	F: AAAGAACTTGGAGGAGGGCG	115
(NM_001285574.1)	R: ACGGCTCTTGTGGAAACCTT	
NF-κB p65	F: CAGCTCACAGATCGGGAAAG	115
(XM_018049265.1)	R: CGGTGCTGTCTGGAAGGAA	
GAPDH	F: TCTGCTGATGCCCCCATGTT	289
(XM_005680968.3)	R: TGACCTTGCCCACAGCCTTG	

## Data Availability

The data that support the findings of this study are available from the corresponding author upon reasonable request.

## Supplementary Materials

The following are available online at https://www.mdpi.com/article/10.3390/ani11092593/s1, Figure S1: Full blots of WB analysis of the conjugated ISG15 after gEECs treated with 5 μg/mL LPS for 0, 3, 6 or 12 h were shown, Figure S2: Full blots of WB analysis of the conjugated ISG15 in LPS-treated gEECs after transducing the si-NC and si-ISG15 were shown. Figure S3: Full blots of WB analysis of the conjugated ISG15 in LPS-treated gEECs after transducing the pcDNA-3.1 and pcDNA-ISG15 were shown.
