# Supplementary figures and images for: ISGylation Inhibits an LPS-Induced Inflammatory Response via the TLR4/NF-κB Signaling Pathway in Goat Endometrial Epithelial Cells
Source: Animals (Basel). 2021 Sep 3;11(9):2593. doi: 10.3390/ani11092593 (PMC8470639; doi:10.3390/ani11092593)

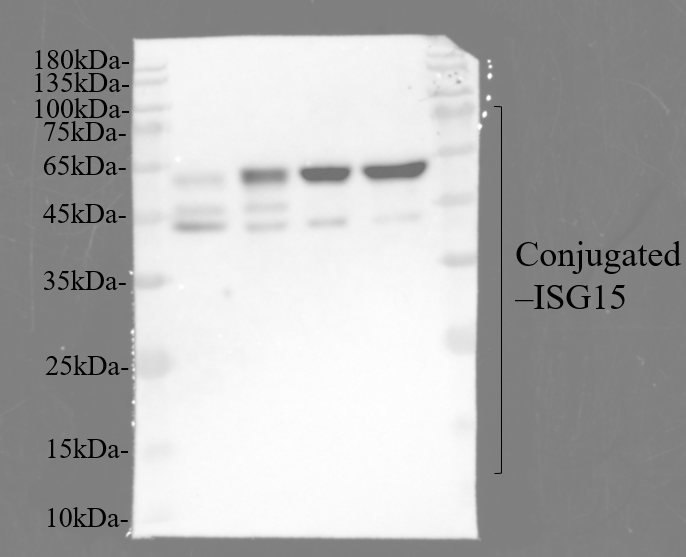

Supplement: Supplementary file 1 [file animals-11-02593-s001.zip › Figure S1.png]

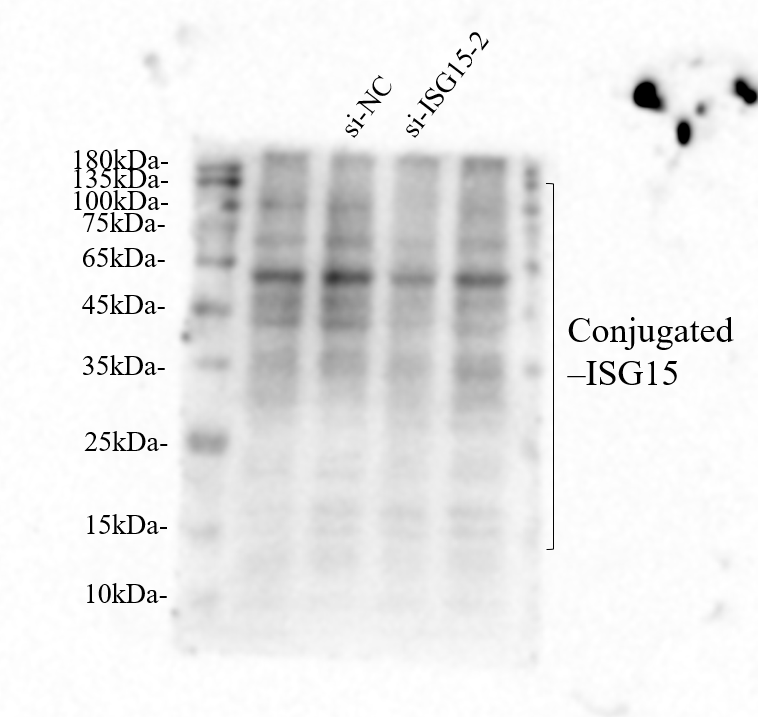

Supplement: Supplementary file 1 [file animals-11-02593-s001.zip › Figure S2.png]

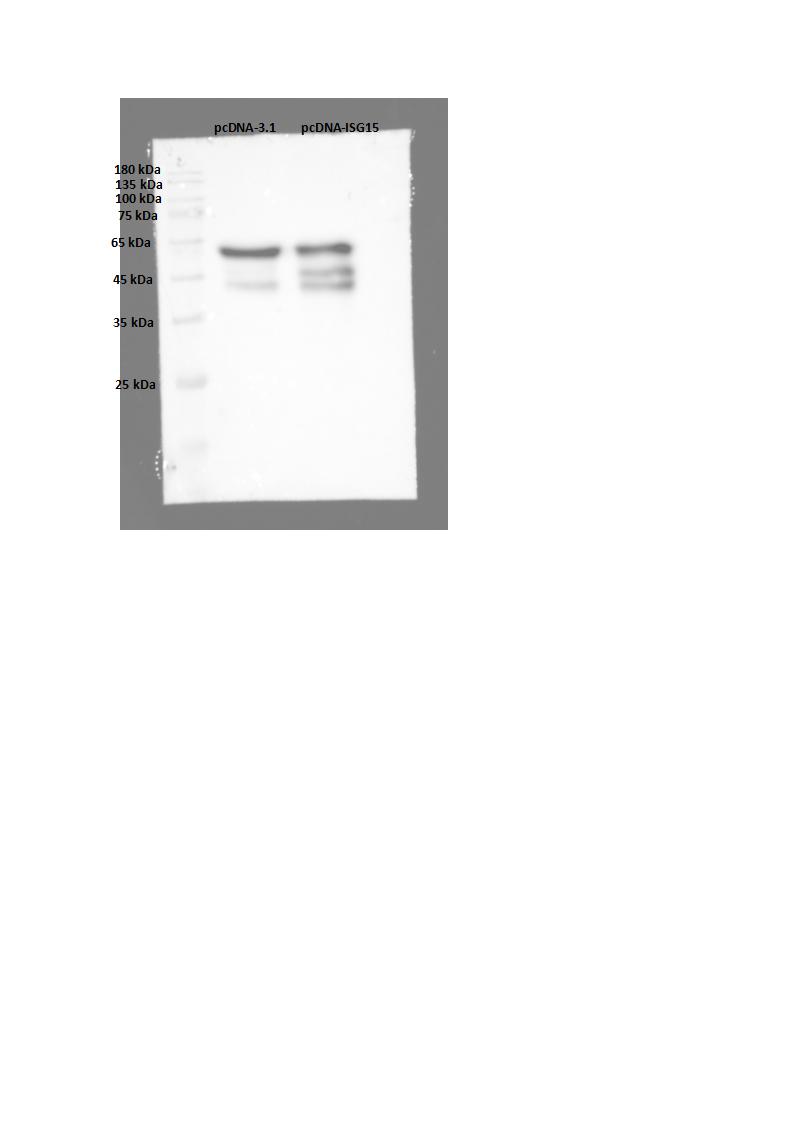

Supplement: Supplementary file 1 [file animals-11-02593-s001.zip › Figure S3.jpg]
